# Notes and News

**Published:** 1889-04-20

**Authors:** 


### Notes and News.

Collected and Communicated.

The next meeting of the Hospitals Association will be held on Tuesday, the 30th inst., at the Westminster Town Hall (Room No. 13) at 8 p.m., when Dr. T. Gilbart-Smith will read a paper on the following subject: "Should out-patients pay?" The Marquis of Carmarthen, M.P., Chairman of the Council, will preside.

The annual report of the Alexandra Hospital for Children at Brighton tells of an increase in the number of both in and out patients, and of the debt to the treasurer as greatly reduced. The bazaar, which was organised by the matron, Mrs. Brown, brought in £100, and one lady who attended it was so struck with the arrangements of the hospital, that she left the institution £1,000 in her will.

The Meath Hospital, Dublin, is asking Government to grant them £600 out of the estate of the late Mr. Matthew O'Reilly Dease, who has left £44,000 for the reduction of the National Debt. Mr. Dease had a real and constant interest in the Meath Hospital, which was very natural, his father having been a president of the Royal College of Surgeons, and his grandfather a surgeon of the Meath, and first president of the Royal College of Surgeons in Ireland. He had commenced certain works of reconstruction in the hospital as a lasting memorial to them, and these were unfinished when he died. But the will was made fifteen years before those works were begun, and from want of funds it is impossible now to complete them. Surely the Government cannot refuse, under these circumstances, to give the £600 prayed for.

The foundation-stone of the new workhouse infirmary fo· Coventry was laid on April 4, in spite of inauspicious weather. Some of the workhouse children near the platform commenced the proceedings by singing the hymn, "O Thou, to whom the sick and dying." Mr. Beamish, an energetic guardian of long standing, laid the stone, and in a subsequent speech referred to the necessity of the institution, owing to the overcrowding in the old workhouse. The new infirmary would be 258ft. long. It would possess every infirmary accommodation; the staircases at the ends were fireproof; and the administrative department would be in the centre, so that the nurses would be at hand when required. Mr. Beamish thought the city might be congratulated on possessing such an infirmary at such a reasonable outlay, especially when they considered that at King's Norton an infirmary containing twenty-eight beds cost £5,200. The site occupied by the new building was formerly covered by a monastery inhabited by the White Friars. A commemorative dinner was held in the evening by the guardians.

On April 6 a sale of work made by the inmates of the Broomhill Home for Incurables at Kirkintilloch, was held at the institution. Sir James King, Provost of Glasgow, opened the proceedings, and there was a large gathering of ladies and gentlemen. It was interesting to see what ingenuity and skill many of the suffering inmates were yet capable of. The receipts amounted to over £100.

M. Chevreul, the eminent French chemist, died at Paris on April 8, at the age of 103. M. Chevreul was a pupil of Vanquelin, whom he succeeded in the Chair of Chemistry at the Museum of Natural History. He was a Fellow of the Royal Society of London, and a Commander of the Legion of Honour. Many of his works on chemistry have been translated into English, and a few years ago he was regarded throughout Europe as the greatest authority in his special line.

The *St. James' Gazette* quotes the following account of a Hindu physical club from a native Indian paper: "The hygenic status of the members is highly satisfactory, and the daily evening exercises under a European soldier appointed by the principal have developed a manual dexterity as well as a fair play of organic juncture; acuteness of optic nerve and a free capacity and a reactionary digestinity has served to render every man with might a trencher man—a redemption from arthmatic proximity has saved many from couching their own knell; and many darkling inquests of prophylactics have become believers of the virtues of physical creed."

The Government enquiry relative to the proposal of the Salford Corporation to borrow £13,160 for the site and erection of an infectious hospital was opened last week. The proposed site is called Ladywell. No infectious case in the building would be placed within 100ft. of the boundary, and the nearest house would be 260ft. away. The estimated cost of the building was £21,000, which would be met by the sale of Wilton Hospital, and the £13,000 now applied for was for the purchase of land. Dr. Arthur Ransome said he considered the site proposed a favourable one for a hospital for 100 to 200 patients. The plot was a large one—13 acres—very high, and the aspect good, facing south and west. The hospital, he was told, was for fevers and not for smallpox, and with proper precautions it could be placed on the site without danger to the people residing in the districts. On the opposite side, it was stated that in the course of a single afternoon 408 persons, residing within a radius of a quarter of a mile of the site, had signed a memorial opposing the scheme. The enquiry was finally adjourned till May 2nd.

One of the late lectures at the Royal Institution was by Mr. A. Gordon Salamon on "Yeast." The lecture was illustrated by magic-lantern slides, and was of considerable interest. Mr. Salamon said that though used for ages there had been but few experiments made as to the nature and action of yeast, with the noted exception of Pasteur's, until quite recently. Pasteur's investigation, however, of its action on sugar would alone have been sufficient to make his scientific reputation. The action of the yeast plant was to convert carbo-hydrates (sugar, etc.) into alcohol and carbonic acid, and further from alcohol to acetic acid (vinegar). The plant, a single cell, was but the 1-3,750th of an inch. The cell subdivisions and growth were rapid, and Pasteur has watched its progress for two hours without leaving the microscope. Hausen, of Copenhagen, had studied the growth with pure cultivating media, and also had succeeded in producing "daughter-cells" different in shape and size from the "mother-cells.' It was, however, for the action of the fungus that man cared, and the question whether shape and size had any influence on its function was now being studied.

The following vacancies are announced this week, the amount of the salary in each case being given after the name of the institution:

**Resident Medical Officers.**

Cheltenham General Hospital. Assistant House Surgeon.—£40 per annum, with board, &c.
Clinical Hospital for Women and Children, Manchester. House Surgeon.—£80 per annum, with board, &c.
Denbighshire Infirmary, Denbigh. House Surgeon.—£85 per annum, with board, &c.
Great Northern Central Hospital, Holloway-road, N. House Physician.—£50 per annum, with board, &c.
Montrose Royal Lunatic Asylum. Junior Assistant Medical Officer.—£100 per annum, with board, &c.
Royal Isle of Wight Infirmary, Ryde. House Surgeon and Secretary.—£50 per annum, with board, &c.
Smedley's Hydropathic Establishment, Malvern. Medical Superintendent.—First year £105, second year £210, with board.
Torbay Hospital and Provident Dispensary, Torbay. Junior House Surgeon and Dispenser.

**Non-Resident Medical Officers.**

Borough of Blackburn. Medical Officer of Health.—£500 per annum.
Bristol General Hospital. Surgeon.
Lampeter Union. Medical Officer and Public Vaccinator. £40 per annum and fees.
Parish of Great Yarmouth. Medical Officer for Health District.—£80 per annum and fees.

A private subscription ball is to be given on the evening of 29th May, at Prince's Hall, Piccadilly, in aid of that very excellent institution the Cottage Hospital, founded in 1872 by Mrs. Black. The dance is under the patronage of their R.H.'s the Prince and Princess Christian, the Princess Louise, Prince and Princess Henry of Battenberg, the Duke and Duchess of Teck, and a very large and fashionable committee. A brilliant gathering may be anticipated, and it is equally safe to expect that a handsome sum will be realised for the Cottage Hospital.

The Dublin Eye and Ear Infirmary received 377 patients during last year—many of the cases requiring very lengthened treatment. In the out-patient department there was an average daily attendance of 51·4 patients. In the Dispensary for Diseases of the Throat, which was established four years ago as an adjunct to the ear department, 167 new patients have been treated during the year. There is a balance in hand of £19 only; this amount should be augmented.

There has been some interesting reading in the report of the Select Committee of the House of Lords appointed to enquire into the sweating system. It has conclusively proved that every business has its special illness, to which those who work at it are more or less liable. The sufferings of the poor chainmakers at Cradley are only of a different form to the sufferings of the matchmakers and tailors and seamstresses. Probably the life of a chainmaker is no more unhealthy than that of any other labourer if the labour is not carried to excess. But it is with this "if" that the evil lies. Dr. Squire, of the North London Hospital for Consumption, said that one quarter of the whole number of deaths from consumption in London were those of tailors. But mere tailoring should kill no man, unless he sat too long over his work and never got fresh air and exercise and wholesome food. This just what the tailors do, and it is therefore not their work which kills them, but the evils of this wretched sweating system which saps all life out of a man, and reduces him beneath the level of the slave. If only some practical laws arise from this enquiry into the sweating system, we shall have less illness in our midst, and more empty beds in our hospitals.

The annual report of the North Riding Infirmary is published. In-patients numbered 694, and out-patients 2,106, as compared with 594 in 1886, and 2,007 in the previous year. Subscriptions and donations were £3,092 8s. 2d., or an increase of £283 3s. on last year's revenue. The expenditure was £2,996 4s. 5d. A ward is closed for want of funds, but as trade is looking up at Middlesbrough this ought to be shortly remedied.

Falkirk Cottage Hospital is nearly completed, and will be opened this summer. It consists, beside the offices in the old building, of a new building in two storeys, with a ward in each—one for male and another for female patients. Each ward measures 34ft. by 20ft., and has a bathroom and necessary conveniences attached to it. The wards are intended to accommodate eight beds each, but are large enough to afford accommodation for ten beds if required. A small auxiliary building has been erected at the west end of the ground for a washing-house and laundry, and also a mortuary. The hospital is not endowed.

The Bishop of Chichester presided lately at an influential gathering of ladies and gentlemen to celebrate the opening of an in-patients' department to the Brighton Throat and Ear Dispensary, thus changing that institution into a hospital. The number of patients who attended the dispensary last year was 1,121, and one-third of the expenses was defrayed from the patients' payments. A large number of the medical men of Brighton have approved this formation of a new hospital, including Sir Tindal Robertson and Dr. Ewart. The matron is Miss Amy Winifred Scott, and she has under her charge two wards, each containing three beds.

Dr. Barry's report of the epidemic of smallpox at Sheffield in 1887 is all in favour of vaccination. Dr. Barry ascertained that 101 among every thousand unvaccinated children, or ten per cent., were seized with smallpox, with a death-rate of 44, while of every thousand vaccinated the number affected was only five, and the death-rate was ·09. Of vaccinated children living in infected houses the rate per thousand who took the disease was 78, and the death-rate 1. Among unvaccinated children in the like case the number per thousand affected was 869, and the death-rate 381. This report should be read before the Royal Commission on vaccination just appointed.

The responsibilities of a Board of Guardians seem to be understood in a rather narrow fashion at Torquay. A woman named Harriet Wilson was admitted to the Torbay Hospital as an urgent case on March the 23rd, and died on the 30th. She had neither relatives nor private means, and the hospital authorities asked Mr. Tozer, the relieving officer for Torquay, to make arrangements for her burial. Mr. Tozer said in a lordly way that his time was too valuable to be wasted in making such arrangements, but that if Mr. Eales, the house surgeon of the hospital—whose time was, presumably, less precious—made the arrangements he would give an order for payment of the costs of the funeral. But when the woman was buried and the hospital authorities asked for payment, Mr. Tozer repudiated all responsibility, as the woman was not in receipt of parish relief when she went into the hospital. O, wise Tozer, if that woman had not been received into the hospital to spend her last hours in comfort and decency, if she had died in a hovel or in the streets, would you not have been compelled to spend your precious time and the parish money in seeing to her burial? Does her admission to a hospital ward make any fundamental change in her circumstances? And seeing how much hospitals do every year to prevent the sick becoming permanently helpless, and consequently unable to earn their living, even a Tozer might, one would think, be brought to see that it is bad economy to use money subscribed for the relief of sickness for the burial of the dead.

